# Effects of Food-Simulating Liquids and Thermocycling on Surface Roughness and Color Stability of Highly Filled Flowable Resin Composites

**DOI:** 10.3390/polym18182196

**Published:** 2026-09-09

**Authors:** Nihat Can Yılmaz, Alper Kaptan

**Affiliations:** Department of Restorative Dentistry, Faculty of Dentistry, Sivas Cumhuriyet University, Sivas 58140, Turkey; alperkapdan@gmail.com

**Keywords:** highly filled flowable resin composites, food-simulating liquids, thermocycling, surface roughness, color stability, resin-based restorative materials

## Abstract

Highly filled flowable resin composites have been developed to improve the mechanical and esthetic performance of conventional flowable materials, but their long-term behavior under different aging conditions remains insufficiently characterized. This study evaluated the effects of thermocycling and food-simulating liquids on the surface roughness and color stability of five highly filled flowable resin composites with a nanohybrid flowable composite included as a reference material. A total of 240 cylindrical specimens were randomly assigned to thermocycling, 10% citric acid, *n*-heptane, or 75% ethanol groups (*n* = 10 per material and protocol). Surface roughness was measured before and after aging, and color change (ΔE*ab) was determined after subsequent immersion in coffee solution. Data were analyzed using two-way analysis of variance (ANOVA) to evaluate the main effects of material and aging protocol and their interaction, followed by Tukey’s post hoc test for multiple comparisons (α = 0.05). Aging-related changes in surface roughness and color stability varied according to both the material and aging protocol. Significant material × aging-protocol interactions were observed for both ΔRa and ΔE*ab (*p* < 0.001). GrandioSO Heavy Flow showed the greatest increase in surface roughness after ethanol and n-heptane aging and the highest mean ΔE*ab values under each aging condition, whereas Neo-Spectra ST Flow showed the highest mean ΔRa after thermocycling, and G-ænial Universal Injectable showed the highest mean ΔRa after citric acid exposure. These findings indicate that the aging response of highly filled flowable resin composites is material-dependent and provide insight into their relative behavior under different laboratory aging conditions.

## 1. Introduction

Resin composites are polymer-based restorative materials consisting primarily of an organic resin matrix, inorganic filler particles, and a coupling agent that promotes adhesion between these phases. Their properties are therefore influenced not only by filler characteristics but also by the composition and structure of the polymer matrix and the integrity of the filler–matrix interface [1,2]. Resin composites are widely used for anterior and posterior direct restorations because of their favorable esthetic properties, adhesive bonding capability, and compatibility with minimally invasive treatment approaches [1,2]. Recent advances in materials science have focused on improving the physical, mechanical, and optical properties of resin composites. In particular, advances in filler technology have enabled the development of restorative materials with improved durability, esthetics, and clinical performance [1,2]. The long-term clinical success of resin composite restorations depends not only on their initial mechanical properties but also on their ability to retain these properties under the biological, chemical, and thermal challenges of the oral environment. Accordingly, surface roughness and color stability are key determinants of the long-term clinical performance of resin composites [3].

Increased surface roughness may promote plaque accumulation, bacterial colonization, surface staining, wear of the opposing dentition, and adverse effects on periodontal tissues [4]. Similarly, impaired color stability, particularly in esthetically demanding regions, may reduce patient satisfaction and necessitate premature restoration replacement [5,6]. Thus, preserving surface integrity and color over time is essential for long-term clinical success [3].

Flowable resin composites are widely used because of their low viscosity, close adaptation to cavity walls, ease of handling, and suitability for minimally invasive restorative procedures [2]. However, in first-generation flowable composites, the lower filler content and higher resin matrix content required to improve flowability compromised mechanical properties, increased polymerization shrinkage, reduced wear resistance, and impaired color stability [1]. Highly filled flowable resin composites were developed to address these limitations. With filler contents of up to approximately 75 wt%, these materials are intended to provide greater mechanical strength, improved wear resistance, lower polymerization shrinkage, and better esthetic performance than conventional flowable resin composites [7]. These improved properties have expanded their potential indications beyond small cavities to posterior restorations and stress-bearing areas [7].

Nevertheless, the oral cavity presents a dynamic and complex environment for restorative materials. During daily function, restorations are exposed to temperature fluctuations from hot and cold foods and beverages, as well as to saliva, acidic and alcoholic drinks, fatty foods, and other chemical agents. These environmental factors may promote resin matrix softening, water sorption, hydrolytic degradation, deterioration of the filler–matrix interface, and eventual filler particle debonding [5,8]. From a polymer-science perspective, these aging processes involve interactions between the polymer network and the surrounding chemical and thermal environment, which may alter the structural integrity and surface behavior of resin-based composites [8,9]. Consequently, surface roughness may increase, while optical properties and color stability may deteriorate [5].

Artificial aging methods are used to simulate the long-term behavior of resin composites in the oral environment. Among these methods, thermocycling and immersion in food-simulating liquids (FSLs) are widely used to reproduce oral thermal and chemical challenges under laboratory conditions [9,10]. Thermocycling simulates oral temperature fluctuations, whereas 75% ethanol, 10% citric acid, and *n*-heptane simulate exposure to alcoholic beverages, acidic foods and fruits, and fatty foods, respectively [11]. These agents may degrade the polymer network, weaken the filler–matrix interface, and impair surface properties [9]. However, the extent of these changes varies with filler type, filler loading, particle size, and resin matrix composition [4].

Although the effects of thermocycling, food-simulating liquids, and beverages have been investigated in conventional nanohybrid and bulk-fill resin composites, evidence on the surface behavior and color stability of highly filled flowable resin composites under these aging conditions remains limited. Comparative studies evaluating several highly filled flowable resin composites within the same experimental model after thermocycling and exposure to multiple food-simulating liquids are particularly scarce [7,12]. Therefore, evaluating these materials from a polymer-based structure–property perspective may help clarify whether aging resistance is primarily associated with high filler loading or with the combined characteristics of the resin matrix, polymer network, and filler–matrix interface [1,2]. Further investigation is therefore needed to characterize the aging behavior of these increasingly used restorative materials.

This study evaluated changes in the surface roughness and color stability of five highly filled flowable resin composites and one nanohybrid flowable resin composite after thermocycling and exposure to different food-simulating liquids. The null hypothesis was that thermocycling and different food-simulating liquids would not produce significant differences in the surface roughness and color stability among the tested resin composites.

## 2. Materials and Methods

### 2.1. Study Design and Ethical Approval

This in vitro study was approved by the Non-Interventional Clinical Research Ethics Committee of Sivas Cumhuriyet University (approval no. 2025-02/33; 20 February 2025). All experimental procedures were performed in the Research Laboratory and the Department of Restorative Dentistry Laboratory, Faculty of Dentistry, Sivas Cumhuriyet University, Sivas, Turkey.

### 2.2. Sample Size Calculation

The sample size was determined by a power analysis conducted by the Department of Biostatistics, Faculty of Medicine, Sivas Cumhuriyet University. The significance level was set at α = 0.05, and statistical power was set at 90% (1 − β = 0.90). The analysis indicated that 40 specimens were required per material, yielding a total sample size of 240 specimens. The post hoc statistical power of the study was calculated to be 0.901.

### 2.3. Resin Composites Used in the Study

Five highly filled flowable resin composites (Clearfil Majesty ES Flow, Omnichroma Flow, G-ænial Universal Injectable, Neo-Spectra ST Flow, and GrandioSO Heavy Flow) and one nanohybrid flowable resin composite (3M Filtek Supreme Flowable), which served as the control, were evaluated. All resin composites were tested in shade A2, except Omnichroma Flow, which was used in its universal single-shade formulation. The composition, filler characteristics, filler loading, shade, manufacturers, and batch numbers of the tested composites are presented in Table 1.

### 2.4. Specimen Preparation

A single calibrated operator prepared and evaluated all specimens to minimize operator-dependent variability. Calibration of the profilometer and spectrophotometer was performed according to the manufacturers’ instructions before each measurement session. Surface roughness and color measurements were obtained in triplicate, and the mean values were used for statistical analysis. Standardized cylindrical specimens measuring 4 mm in diameter and 2 mm in height were fabricated using Teflon molds. The composites were placed into the molds carefully to minimize air entrapment. A transparent Mylar strip and glass slide were placed over the material, and gentle pressure was applied to remove excess composite and produce a flat surface [13].

All specimens were light-cured for 20 s from each surface, for a total of 40 s, using a light-emitting diode (LED) curing unit (Woodpecker I-Led II, Guilin Woodpecker Medical Instrument Co., Ltd., Guilin, China; irradiance, 2300 mW/cm^2^; wavelength range, 420–480 nm). The light-curing protocol (20 s per surface) was selected in accordance with the manufacturers’ instructions for all tested resin composites. After polymerization, the specimens were stored in distilled water at 37 °C for 24 h to allow post-curing polymerization.

To standardize the surfaces, all specimens were wet-ground with 1000-grit silicon carbide abrasive paper [14]. The specimens were then polished with fine (20 μm) and extra-fine (10 μm) OptiDisc polishing disks (Kerr Corporation, Orange, CA, USA) according to the manufacturer’s instructions.

For each material, the specimens were allocated to four aging subgroups (*n* = 10 per subgroup) corresponding to the following aging protocols:

Group 1: Thermocycling;
Group 2: 10% citric acid;
Group 3: *n*-heptane;
Group 4: 75% ethanol.

### 2.5. Surface Roughness Measurements

Baseline surface roughness was measured using a contact profilometer (Surftest SJ-301, Mitutoyo Corp., Kawasaki, Japan) equipped with a 5-μm-radius diamond stylus. Measurements were performed using an applied force of 0.75 mN, a tracing speed of 0.5 mm/s, a tracing length of 4 mm, and a cutoff value of 0.8 mm in accordance with ISO 4287/4288 standards [15,16]. For each specimen, three parallel measurements were performed over the central surface area under standardized conditions, and the mean of the three measurements was recorded as the baseline arithmetic mean surface roughness (Ra) for each specimen. After the aging procedures, surface roughness was measured again using the same measurement protocol over the central surface area. The exact scan paths were not predefined or reproduced between baseline and post-aging measurements.

### 2.6. Color Measurements

Color was measured using a Vita Easyshade Advance spectrophotometer (VITA Zahnfabrik, Bad Säckingen, Germany) according to the Commission Internationale de l’Éclairage L*a*b* (CIELAB) color system [17]. In the CIELAB color space, L* represents lightness, a* represents the red-green chromatic coordinate, and b* represents the yellow-blue chromatic coordinate. The spectrophotometer was calibrated before each measurement session according to the manufacturer’s instructions and recalibrated after every three specimens. All measurements were performed against a white background under standardized conditions. Each specimen was positioned consistently, and three consecutive measurements were obtained from the center of each specimen. The mean L*, a*, and b* values were recorded for statistical analysis. To minimize environmental variation, all measurements were performed at the same location and at the same time of day throughout the study.

### 2.7. Aging Protocols

The conditions used for each aging protocol are summarized in Table 2.

The aging protocols were selected to represent different thermal and chemical challenges that resin composites may encounter in the oral environment. Thermocycling was used to simulate repeated intraoral temperature fluctuations, whereas the food-simulating liquids represented different dietary chemical exposures: 10% citric acid represented acidic foods and beverages, *n*-heptane represented fatty food components, and 75% ethanol represented alcohol-containing substances [11]. These protocols were intended as standardized laboratory models of distinct aging challenges rather than direct simulations of specific clinical exposure durations.

### 2.8. Post-Aging Evaluations

After the aging procedures, surface roughness was remeasured using the same contact profilometer and the same measurement parameters as those used for the baseline measurements. For each specimen, three parallel measurements were performed over the central surface area, and the mean Ra value was used for statistical analysis. The change in surface roughness (ΔRa) was calculated as the difference between the post-aging and baseline Ra values (ΔRa = post-aging Ra − baseline Ra), with positive values indicating an increase in surface roughness after aging.

To evaluate color stability, each specimen was immersed individually in 30 mL of a freshly prepared coffee solution in a 100 mL specimen container at room temperature for 24 h. The coffee solution was prepared by dissolving 2 g of Nescafé Classic in 200 mL of boiling distilled water. A fresh coffee solution was prepared immediately before staining each experimental group. After immersion, the specimens were rinsed thoroughly with distilled water to remove residual coffee deposits, gently air-dried until no visible surface moisture remained, and immediately remeasured under the same standardized color measurement conditions. Color change (ΔE*ab) was calculated using the conventional CIELAB (1976) color-difference formula based on the L*, a*, and b* coordinates, as follows:

$${\Delta E}_{ab}^{*}\;=\;\sqrt{\left[{\left(L_{1}\;-\;L_{0}\right)}^{2}\;+\;{\left(a_{1}\;-\;a_{0}\right)}^{2}\;+\;{\left(b_{1}\;-\;b_{0}\right)}^{2}\right]}$$

Here, *L*_0_, *a*_0_, and *b*_0_ represent the baseline color coordinates measured before the aging procedures, whereas *L*_1_, *a*_1_, and *b*_1_ represent the color coordinates measured after completion of the aging protocol and subsequent coffee immersion. Therefore, ΔE*ab represents the overall color change resulting from both the aging procedure and coffee staining.

### 2.9. Statistical Analysis

Statistical analyses were performed using IBM SPSS Statistics software (Version 23.0; IBM Corp., Armonk, NY, USA). The normality of the data distribution was assessed using the Shapiro–Wilk test, and homogeneity of variances was evaluated using Levene’s test. Two-way analysis of variance (ANOVA) was performed for ΔRa and ΔE*ab to evaluate the main effects of resin composite material and aging protocol, as well as the material × aging-protocol interaction. Given the significant interaction effects, Tukey’s post hoc test was used for multiple comparisons among materials within each aging protocol and among aging protocols within each material. The association between ΔRa and ΔE*ab was examined using Pearson’s correlation analysis, with the individual specimen as the unit of analysis (*n* = 240). All statistical tests were two-sided, and a *p*-value < 0.05 was considered statistically significant.

## 3. Results

The two-way ANOVA results for ΔRa and ΔE*ab are summarized in Table 3. Significant material × aging-protocol interactions were observed for both outcomes (*p* < 0.001).

### 3.1. Changes in Surface Roughness (ΔRa)

**Figure 1:**
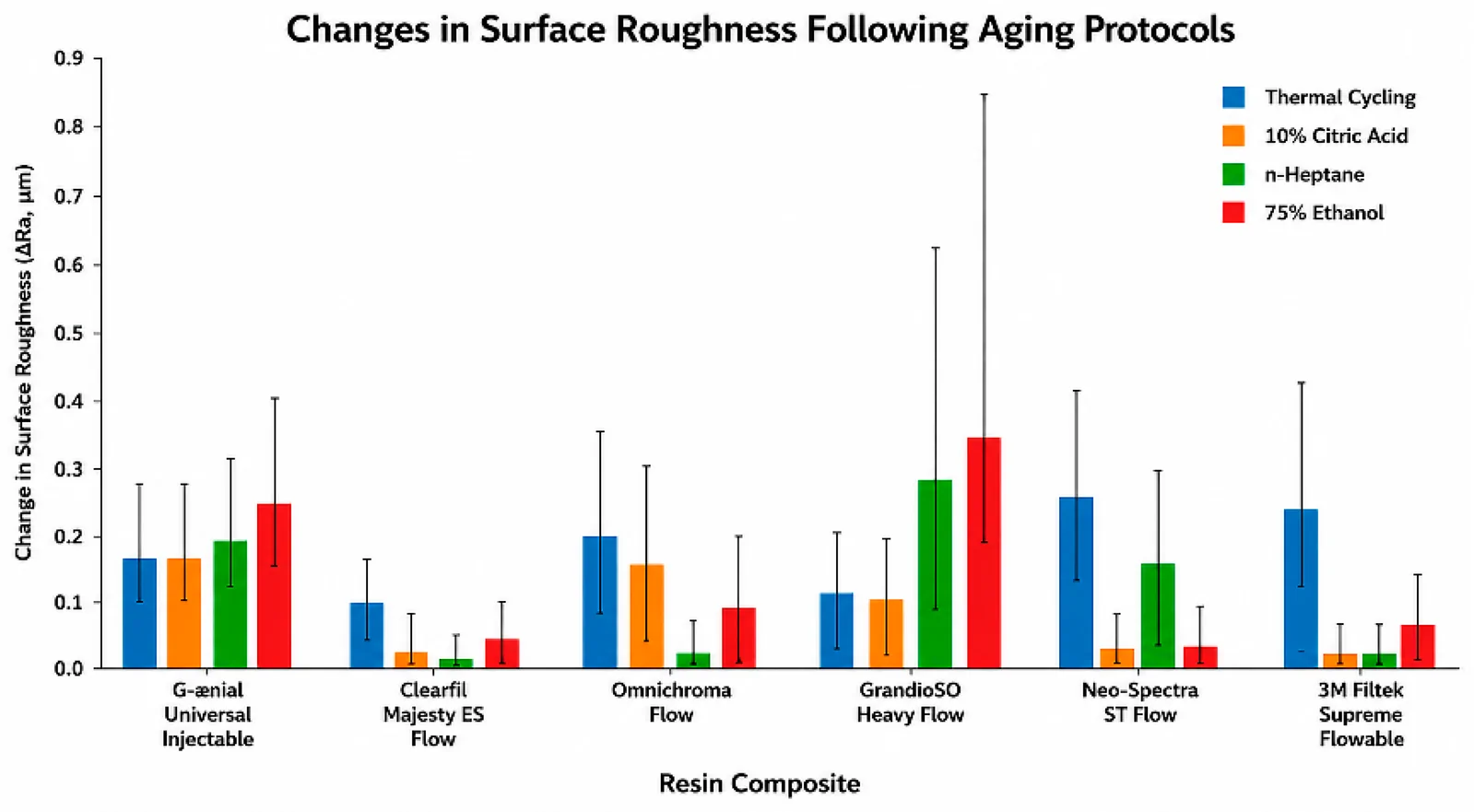
Effect of different aging protocols on the change in surface roughness (ΔRa, µm) of the investigated resin composites. Positive ΔRa values indicate an increase in surface roughness, whereas negative ΔRa values indicate a decrease. Error bars represent standard deviation (SD).

The mean changes in surface roughness (ΔRa) after the aging protocols are presented in Table 4 and Figure 1.

Two-way ANOVA revealed a significant main effect of aging protocol on ΔRa (F(3, 216) = 9.273, *p* < 0.001), whereas the main effect of resin composite material was not statistically significant (F(5, 216) = 2.239, *p* = 0.052). A significant material × aging-protocol interaction was observed (F(15, 216) = 4.243, *p* < 0.001), indicating that the effect of aging protocol on surface roughness varied according to the resin composite material.

ΔRa differed significantly among the aging protocols for Omnichroma Flow, GrandioSO Heavy Flow, Neo-Spectra ST Flow, and 3M Filtek Supreme Flowable (*p* < 0.05), whereas no significant differences were observed for G-ænial Universal Injectable or Clearfil Majesty ES Flow (*p* > 0.05; Table 4).

Among the aging protocols, GrandioSO Heavy Flow showed the highest mean ΔRa after exposure to 75% ethanol and *n*-heptane. Neo-Spectra ST Flow showed the highest mean ΔRa after thermocycling, whereas G-ænial Universal Injectable showed the highest mean ΔRa after exposure to 10% citric acid (Table 4).

### 3.2. Color Change (ΔE*ab)

**Figure 2:**
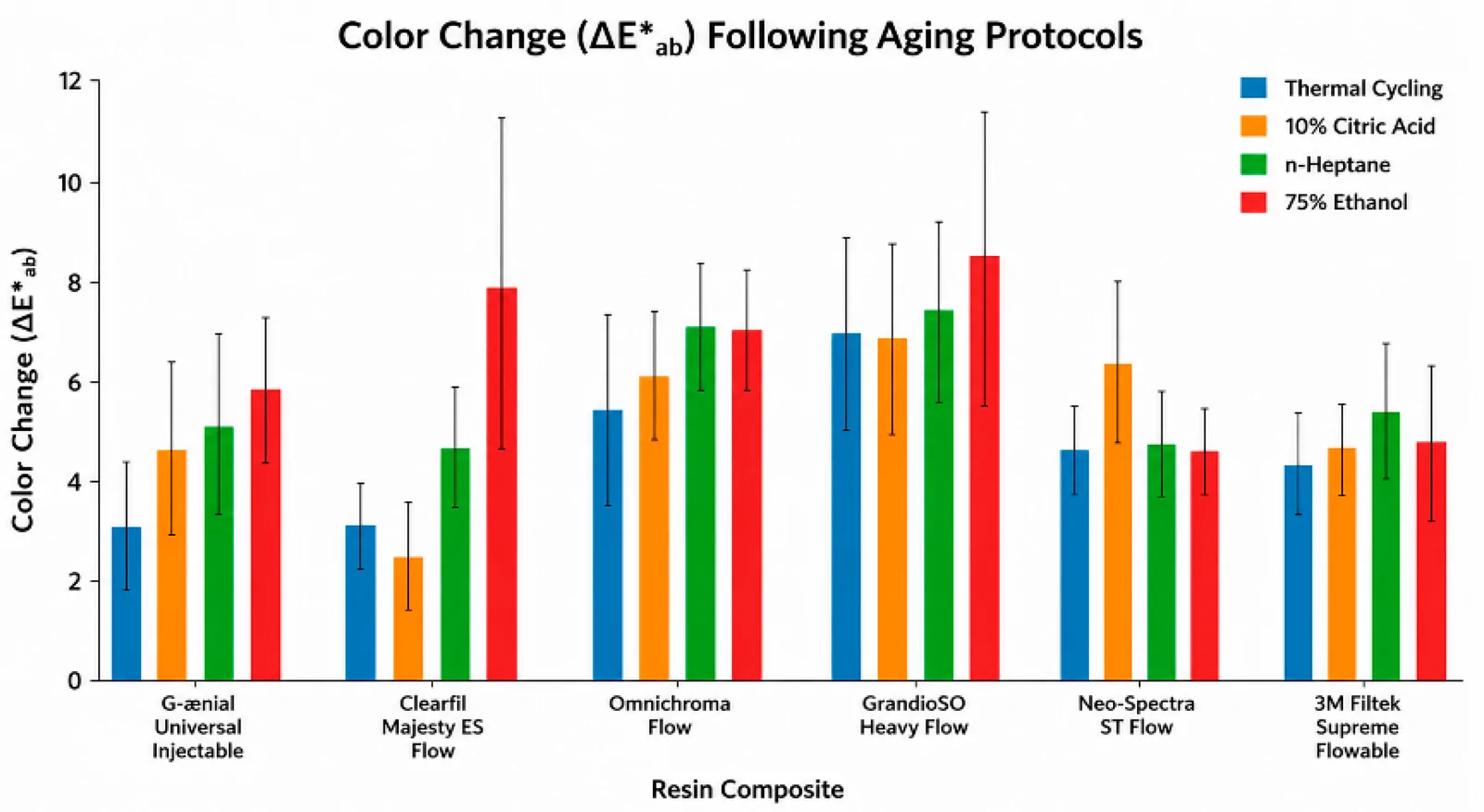
Effect of different aging protocols on the color change (ΔE*ab) of the investigated resin composites. Error bars represent standard deviation (SD).

The mean color changes (ΔE*ab) after coffee immersion following each aging protocol are presented in Table 5 and Figure 2.

For ΔE*ab, two-way ANOVA revealed significant main effects of resin composite material (F(5, 216) = 21.144, *p* < 0.001) and aging protocol (F(3, 216) = 15.400, *p* < 0.001). A significant material × aging-protocol interaction was also observed (F(15, 216) = 4.454, *p* < 0.001), indicating that the effect of aging protocol on color change varied according to the resin composite material.

ΔE*ab differed significantly among the aging protocols for G-ænial Universal Injectable, Clearfil Majesty ES Flow, and Neo-Spectra ST Flow (*p* < 0.05), whereas no significant differences were observed for Omnichroma Flow, GrandioSO Heavy Flow, or 3M Filtek Supreme Flowable (*p* > 0.05; Table 5).

Overall, GrandioSO Heavy Flow showed the highest mean ΔE*ab values among the tested materials. In particular, GrandioSO Heavy Flow specimens aged in 75% ethanol showed the highest mean ΔE*ab value (Table 5).

### 3.3. Relationship Between Surface Roughness and Color Change

Pearson’s correlation analysis revealed no statistically significant association between changes in surface roughness (ΔRa) and color change (ΔE*ab) (*r* = 0.076, *p* = 0.240; *n* = 240).

## 4. Discussion

This study compared the effects of different aging protocols on the surface roughness and color stability of highly filled flowable resin composites. Both thermocycling and food-simulating liquids produced changes in all tested materials, although the magnitude of these changes varied among the resin composites. The aging response also varied with the aging protocol and material composition, including the monomer system, filler type, filler loading, and filler–matrix interfacial characteristics. The significant material × aging-protocol interactions observed for both ΔRa and ΔE*ab further demonstrate that the effects of aging were dependent on the resin composite material. Therefore, the null hypothesis was rejected, as significant differences in surface roughness and color stability were observed depending on the resin composite material and aging protocol. One of the principal findings of this study was that ethanol and *n*-heptane produced greater changes in surface roughness in some highly filled flowable resin composites, whereas materials such as Clearfil Majesty ES Flow exhibited relatively stable surface roughness values under the tested aging conditions. These findings suggest that the aging resistance of highly filled flowable resin composites is not determined solely by filler loading and may also depend on resin matrix composition, polymer network characteristics, and filler–matrix interfacial stability [1,2].

### 4.1. Surface Roughness

Surface roughness is an important determinant of the long-term clinical performance of restorative materials. Increased surface roughness may promote undesirable clinical consequences, including plaque accumulation, bacterial adhesion, surface staining, increased wear of the opposing dentition, and deterioration of restoration esthetics [4]. Evaluating the surface behavior of resin composites under different aging conditions may therefore help estimate their long-term performance.

In this study, the significant material × aging-protocol interaction for ΔRa demonstrated that the effects of the aging protocols on surface roughness were material-dependent. Significant differences among aging protocols were observed only for Omnichroma Flow, GrandioSO Heavy Flow, Neo-Spectra ST Flow, and 3M Filtek Supreme Flowable, whereas G-ænial Universal Injectable and Clearfil Majesty ES Flow showed no statistically significant differences. More pronounced surface alterations were observed in some resin composites following aging in ethanol and *n*-heptane, whereas the effects of thermocycling and citric acid varied according to the material composition [18]. These findings are consistent with previous reports indicating that food-simulating liquids can adversely affect the physical properties of resin composites. McKinney and Wu suggested that organic solvents may soften the resin matrix and promote degradation at the filler–matrix interface [19]. Similarly, Kooi et al. reported increased surface roughness after chemical aging of resin composites [10].

One of the most notable findings of this study was that GrandioSO Heavy Flow showed the highest mean ΔRa values, particularly following aging in ethanol and *n*-heptane. Although materials with higher filler loading are generally expected to exhibit greater resistance to degradation, the present findings suggest that filler loading alone may not determine surface stability. Rather, surface degradation is a multifactorial phenomenon and may also be influenced by differences in resin matrix composition, filler morphology, silanization quality, and the hydrolytic stability of the filler-matrix interface [1,2,20,21].

Ethanol may penetrate the polymer network and plasticize the resin matrix. In Bis-GMA- and TEGDMA-containing resin systems, solvent absorption may increase intermolecular spacing, reduce cross-link density, and soften the resin matrix by disrupting polymer-network interactions. These changes may promote microvoid formation, weaken the filler-matrix interfacial bond, and increase the likelihood of filler particle loss. Sideridou et al. reported that water uptake may contribute to hydrolytic degradation of the filler–matrix interface [8]. Likewise, Voltarelli et al. and Kumari et al. identified ethanol as a particularly aggressive chemical-aging medium for resin composites [3,22]. The higher mean ΔRa values observed for GrandioSO Heavy Flow following ethanol aging in the present study are consistent with these degradation mechanisms, although the underlying mechanisms were not directly investigated in this study.

The findings for the *n*-heptane group may be interpreted in a similar manner. *n*-Heptane is a nonpolar solvent commonly used to simulate the chemical effects of exposure to fatty foods in the oral environment. Because of its nonpolar nature, *n*-heptane may interact preferentially with the hydrophobic components of the resin matrix and may affect polymer-network stability. The comparatively higher mean ΔRa values observed for GrandioSO Heavy Flow following *n*-heptane aging are consistent with the possibility that solvent–matrix interactions may have contributed to reduced surface integrity [23]. However, these interactions were not directly evaluated in the present study.

The findings following thermocycling were also generally consistent with previous reports. Repeated temperature fluctuations may generate interfacial stresses because the resin matrix and inorganic filler particles have different coefficients of thermal expansion. Over time, such stresses may promote microcrack formation, facilitate water penetration, and contribute to hydrolytic degradation [24]. Ferracane reported that hydrolytic aging can impair polymer-network integrity and surface properties by altering the polymer network structure [25]. Similarly, Yilmaz and Sadeler reported increased surface roughness of resin composites following thermocycling [26]. Although G-ænial Universal Injectable showed comparatively higher mean ΔRa values following thermocycling than after the other aging protocols, these differences were not statistically significant. Nevertheless, this trend is consistent with previous reports describing the potential effects of thermocycling on resin-composite surface characteristics.

Another noteworthy finding of this study was the relatively limited change in surface roughness observed for Clearfil Majesty ES Flow across the tested aging protocols. No statistically significant differences in ΔRa were observed among the aging protocols for this material. This behavior may reflect relatively stable interactions between its resin matrix and silanized barium-glass fillers. A stable filler-matrix interface may also reduce solvent penetration and filler loss, thereby helping to preserve surface integrity. Similarly, Checchi et al. reported that filler characteristics and polymerization-related factors may influence the surface roughness of resin composites [27]. Although these characteristics were not directly evaluated in the present study, they may partly explain the relatively stable surface roughness observed for Clearfil Majesty ES Flow.

Overall, the findings of this study indicate that the aging behavior of the tested highly filled flowable resin composites was material-dependent. The present results also suggest that high filler loading alone does not necessarily guarantee superior surface stability. Rather, the aging-related surface changes observed in this study should be interpreted in relation to the combined influence of resin matrix composition, polymer-network characteristics, filler characteristics, filler-matrix interfacial integrity, and solvent-polymer interactions. Although these factors provide plausible explanations for the observed findings, they were not directly evaluated in the present study.

### 4.2. Color Stability

Color stability is a key determinant of the long-term esthetic performance of resin composite restorations. Restorative materials are expected to provide an acceptable initial color match and maintain it under the physical and chemical challenges of the oral environment [28]. Color change in resin composites is influenced by resin matrix composition, filler type and loading, water sorption, degree of conversion, surface integrity, and penetration of extrinsic pigments [6,29]. Accordingly, color stability reflects both the esthetic performance and long-term durability of restorative materials [1,5,30].

In this study, the significant material × aging-protocol interaction for ΔE*ab demonstrated that the effects of thermocycling and food-simulating liquids on color stability were material-dependent. Across all aging protocols, GrandioSO Heavy Flow showed the highest mean ΔE*ab values, whereas Clearfil Majesty ES Flow and Neo-Spectra ST Flow exhibited comparatively lower mean ΔE*ab values. These findings suggest that color stability cannot be explained solely by filler loading. Instead, differences in resin matrix composition, monomer system, and the chemical stability of the filler-matrix interface may also have contributed to the observed discoloration behavior of the resin composites [2,31].

Following the aging procedures, all specimens were immersed in a coffee solution to evaluate staining susceptibility. Coffee is one of the most commonly used staining media in laboratory studies because of its low molecular weight yellow pigments and high staining potential [28]. These pigments can diffuse into the resin matrix and produce persistent discoloration, making coffee a useful model for evaluating the staining susceptibility of resin-based restorative materials. Patel et al. and Ertas et al. reported that coffee produces greater discoloration of resin composites than several other commonly consumed beverages after exposure [6,32]. Consistent with these reports, the color changes observed after aging and subsequent coffee immersion support the use of coffee as a clinically relevant staining medium.

Water sorption is considered a major contributor to discoloration in resin composites. Water absorbed by the resin matrix can diffuse between polymer chains, increase intermolecular spacing, promote matrix swelling, and facilitate microvoid formation. These structural changes may facilitate the penetration of extrinsic pigments into the material and reduce color stability [25]. Bagheri et al. and Arocha et al. identified water sorption as an important contributor to pigment diffusion and subsequent discoloration of resin composites [5,30]. Thus, aging-related chemical changes may adversely affect both the surface characteristics and the optical properties of resin composites [25].

GrandioSO Heavy Flow showed the highest mean ΔE*ab values across all aging conditions. This finding may be related to its resin matrix composition. GrandioSO Heavy Flow contains a Bis-GMA- and TEGDMA-based monomer system. The relatively hydrophilic character of TEGDMA may increase water sorption, facilitate pigment penetration, and promote discoloration [8]. Previous studies by Guler et al., Ardu et al., and Fonseca et al. similarly indicated that resin composition may influence the color stability of resin-based materials following aging [33,34,35]. In addition, Redwan [36] reported relatively high ΔE*ab values for materials in the Grandio product family after coffee staining. The present findings are consistent with these previous reports. However, the mechanisms underlying the observed discoloration were not directly investigated in the present study.

By contrast, Clearfil Majesty ES Flow and Neo-Spectra ST Flow showed comparatively lower mean ΔE*ab values. This behavior may reflect differences in resin matrix chemistry and filler-matrix interfacial stability. UDMA-based resin systems have been reported to exhibit lower water sorption than some Bis-GMA-based materials, potentially limiting pigment penetration [8]. Furthermore, effective silanization may improve filler-matrix interfacial stability and resistance to hydrolytic degradation [25]. The present findings suggest that color stability should be interpreted in relation not only to filler loading but also to monomer composition, resin matrix characteristics, and filler-matrix interfacial integrity. Although these characteristics were not directly evaluated in the present study, they may partly explain the comparatively lower ΔE*ab values observed for these materials.

Among the aging protocols evaluated, the highest mean ΔE*ab values were generally observed after aging in 75% ethanol. Ethanol is known to penetrate the polymer network and plasticize the resin matrix [25]. This process may facilitate water sorption and the diffusion of extrinsic pigments into the material. Yap et al., Hahnel et al., and Alshali et al. similarly reported adverse effects of ethanol exposure on the properties of resin-based restorative materials [37,38,39]. The higher mean ΔE*ab values observed in the ethanol groups in the present study are consistent with these previous reports, although the underlying mechanisms were not directly investigated in this study.

Overall, the findings of this study indicate that the color stability of the tested highly filled flowable resin composites is influenced by multiple interacting factors rather than filler loading alone. Monomer composition, water-sorption potential, polymer-network stability, and filler-matrix interfacial integrity may collectively contribute to the extent of discoloration [5,25,28]. Accordingly, in addition to achieving an acceptable initial esthetic appearance, the long-term color stability of restorative materials under relevant chemical-aging conditions should also be considered during material selection [31]. Although these factors provide plausible explanations for the observed findings, they were not directly evaluated in the present study.

### 4.3. Relationship Between Surface Roughness and Color Stability

Although the relationship between surface roughness and color stability has been widely investigated, the strength of this association remains uncertain. Several studies suggest that increased surface roughness facilitates pigment retention and promotes discoloration [14,40]. By contrast, other studies indicate that color change cannot be explained by surface topography alone and is also influenced by the chemical characteristics of the restorative material [28,41].

In the present study, although some materials showed concurrent increases in surface roughness (ΔRa) and color change (ΔE*ab), Pearson’s correlation analysis revealed no statistically significant association between these variables (*r* = 0.076, *p* = 0.240). This finding suggests that color change cannot be explained by surface roughness alone and is likely influenced by multiple interacting material-related factors.

Increased surface roughness may facilitate the retention of staining pigments on the material surface. However, discoloration is not solely a surface phenomenon. Pigment diffusion into the resin matrix, monomer-dependent water sorption, the chemical stability of the polymer network, filler–matrix interfacial integrity, and hydrolytic degradation during aging may all contribute to the overall color change [28,42,43]. Consequently, some materials may show substantial discoloration despite relatively smooth surfaces, whereas others may exhibit only limited color change despite measurable increases in surface roughness [43,44].

In the present study, GrandioSO Heavy Flow exhibited comparatively high ΔRa and ΔE*ab values. Nevertheless, when all materials were analyzed collectively, Pearson’s correlation analysis revealed no statistically significant association between these two parameters. This finding suggests that factors related to material composition, in addition to surface topography, may contribute to color change. Although this result differs from the positive associations reported by Freitas et al. and Meniawi et al. [44,45], it is consistent with the findings of Villalta et al. and Çalışkan et al., who emphasized the multifactorial nature of resin composite discoloration [28,41].

Overall, these findings indicate that surface roughness alone is not a sufficient predictor of the long-term esthetic performance of highly filled flowable resin composites. Evaluating surface characteristics, monomer composition, water sorption behavior, and color stability together may provide a more comprehensive assessment of the performance of these restorative materials.

### 4.4. Clinical Implications

The findings of the present study may have important clinical implications for the selection of highly filled flowable resin composites in restorative practice. Because restorative materials are continuously exposed to thermal fluctuations and chemical challenges in the oral environment, long-term material selection should consider surface integrity and color stability in addition to their initial mechanical and esthetic properties.

The present findings indicate that the aging behavior of highly filled flowable resin composites is material-dependent. Despite their high filler loadings, the materials differed in their resistance to aging, which may be related to differences in resin matrix composition and filler–matrix interfacial stability. The material-dependent responses observed under different aging conditions provide information on the relative behavior of the tested resin composites under specific laboratory challenges. However, these in vitro findings should not be directly extrapolated to material-selection recommendations based on individual dietary habits, and clinical studies are required to confirm their clinical relevance.

Furthermore, the comparatively stable surface behavior of Clearfil Majesty ES Flow under the tested aging conditions suggests that differences in material composition may influence resistance to chemical aging. These findings may help inform material selection when resistance to chemical challenges and esthetic stability are relevant considerations. However, because the present study was conducted under controlled laboratory conditions, the findings should be interpreted with appropriate clinical caution.

### 4.5. Strengths and Limitations

This study has several strengths. A standardized experimental protocol was applied throughout all stages of specimen preparation, finishing and polishing, aging, and outcome assessment to minimize methodological variability. In addition, five commercially available highly filled flowable resin composites and one nanohybrid flowable resin composite, which served as the reference material, were evaluated under identical experimental conditions using four different aging protocols, allowing a systematic comparison of their surface roughness and color stability. The combined assessment of both surface and optical properties may provide a more comprehensive understanding of the behavior of these restorative materials under simulated aging conditions.

This study also has several limitations. First, as an in vitro study, the experimental design could not fully reproduce the complex biological, chemical, and mechanical conditions of the oral environment. Salivary proteins, biofilm formation, masticatory forces, oral hygiene practices, and individual dietary habits may all influence the long-term behavior of restorative materials.

Second, only one shade of each resin composite was evaluated. Therefore, the findings may not necessarily apply to other shades of the same material, as differences in pigment composition and translucency may affect color stability and staining susceptibility.

Third, only three food-simulating liquids, thermocycling, and a single staining solution (coffee) were evaluated. Other commonly consumed beverages and food-simulating liquids may produce different effects on surface degradation and color stability. Therefore, the findings should not be directly extrapolated to clinical conditions.

Fourth, the aging protocols used in this study did not include mechanical loading, toothbrushing abrasion, biofilm formation, or repeated dietary exposure, all of which may influence the long-term clinical performance of restorative materials. Future studies incorporating these factors may provide a more comprehensive simulation of the oral environment.

Fifth, although several mechanisms potentially contributing to surface degradation and discoloration were discussed, including water sorption, hydrolytic degradation, and filler–matrix interfacial changes, these mechanisms were not directly evaluated in the present study. Therefore, these explanations should be interpreted as potential mechanisms rather than experimentally confirmed findings.

### 4.6. Future Perspectives

Future in vivo studies and randomized clinical trials are needed to evaluate the long-term clinical performance of highly filled flowable resin composites under clinical conditions. In addition, laboratory studies should incorporate mechanical loading, toothbrushing simulation, pH cycling, repeated dietary exposure, and biofilm formation to better reproduce the complex conditions of the oral environment.

Further research should investigate the influence of monomer composition, filler morphology, filler surface treatment, and polymer network characteristics on the aging behavior of these materials. Because these factors were not directly evaluated in the present study, such investigations would provide a better understanding of the mechanisms underlying surface degradation and discoloration. Ultimately, these findings may support the development of restorative materials with improved long-term esthetic and clinical performance.

## 5. Conclusions

Within the limitations of this in vitro study, the following conclusions can be drawn:

- Significant material × aging-protocol interactions were observed for both surface roughness and color change, demonstrating that the effects of aging were material-dependent.
- Clearfil Majesty ES Flow exhibited relatively stable surface roughness and color stability under the tested aging conditions.
- GrandioSO Heavy Flow showed comparatively high increases in both surface roughness and color change, particularly after ethanol and *n*-heptane exposure, suggesting greater susceptibility to aging under these conditions.
- No statistically significant association was found between changes in surface roughness (ΔRa) and color change (ΔE*ab), indicating that color change cannot be explained by surface roughness alone.
- The observed material-dependent responses provide information on the relative resistance of the tested resin composites to thermal and chemical aging under laboratory conditions.

## Figures and Tables

**Table 1:** Composition and characteristics of the resin composites used in this study.

Resin Composite	Organic Matrix	Filler Composition	Filler Loading (wt%/vol%)	Shade	Manufacturer (City, Country)	Batch No.
G-ænial Universal Injectable	UDMA, Bis-MEPP, TEGDMA	Silica (16 nm), strontium glass (200 nm), photoinitiators, pigments	69/45	A2	GC (Tokyo, Japan)	2406191
Clearfil Majesty ES Flow	TEGDMA, UDMA, hydrophobic aromatic dimethacrylate	Silanated barium glass, silanated silica, camphorquinone, initiators, accelerators, pigments (0.18–3.5 μm)	75/59	A2	Kuraray Noritake Dental (Okayama, Japan)	850445
Omnichroma Flow	UDMA, 1,9-nonanediol dimethacrylate	Supra-nano spherical SiO_2_–ZrO_2_ fillers (260 nm)	70/57	Universal	Tokuyama Dental (Tokyo, Japan)	101E84
GrandioSO Heavy Flow	Bis-GMA, TEGDMA, HEDMA	Functionalized SiO_2_ nanoparticles and glass ceramic fillers	80/65.6	A2	VOCO (Cuxhaven, Germany)	2440116
Neo-Spectra ST Flow	Modified polysiloxane dimethacrylate resin matrix	Pre-polymerized SphereTEC fillers (d50 ≈ 15 μm), barium glass, ytterbium fluoride	62.5/40	A2	Dentsply Sirona (Konstanz, Germany)	2411000541
3M Filtek Supreme Flowable	Procrylate, Bis-GMA, TEGDMA	Silica (20 nm) and zirconia nanoparticles (4–11 nm)	65/46	A2	3M ESPE (St. Paul, MN, USA)	11295507

Abbreviations: Bis-GMA, bisphenol A glycidyl methacrylate; Bis-MEPP, bisphenol A ethoxylated dimethacrylate; HEDMA, hydroxyethyl dimethacrylate; TEGDMA, triethylene glycol dimethacrylate; UDMA, urethane dimethacrylate.

**Table 2:** Experimental conditions of the aging protocols.

Aging Protocol	Storage Medium	Experimental Conditions
Thermocycling	Distilled water	5000 cycles; 5–55 °C; dwell time: 30 s; transfer time: 5 s
10% citric acid	10% citric acid solution	Stored at 37 °C for 28 days
*n*-Heptane	*n*-Heptane solution	Stored at 37 °C for 28 days
75% ethanol	75% ethanol solution	Stored at 37 °C for 28 days

Abbreviations: °C, degrees Celsius; s, seconds.

**Table 3:** Two-way ANOVA results for changes in surface roughness (ΔRa) and color change (ΔE*ab).

Outcome	Effect	df	F	*p*-Value
ΔRa	Resin composite material	5, 216	2.239	0.052
	Aging protocol	3, 216	9.273	<0.001 *
	Material × aging protocol	15, 216	4.243	<0.001 *
ΔE*ab	Resin composite material	5, 216	21.144	<0.001 *
	Aging protocol	3, 216	15.400	<0.001 *
	Material × aging protocol	15, 216	4.454	<0.001 *

* Statistically significant at *p* < 0.05.

**Table 4:** Mean changes in surface roughness (ΔRa) after the aging protocols (mean ± SD).

Resin Composite	Thermocycling	10% Citric Acid	*n*-Heptane	75% Ethanol
G-ænial Universal Injectable	0.00 ± 0.18 ^A,a^	0.17 ± 0.20 ^A,a^	0.17 ± 0.16 ^A,a^	0.24 ± 0.39 ^A,c,d^
Clearfil Majesty ES Flow	0.09 ± 0.28 ^A,a,b^	0.00 ± 0.20 ^A,a,b^	0.00 ± 0.08 ^A,a,c,d^	0.04 ± 0.20 ^A,c,d^
Omnichroma Flow	0.19 ± 0.10 ^A,b^	0.15 ± 0.25 ^B,b^	0.01 ± 0.08 ^A,B,a^	0.08 ± 0.13 ^B,a,d^
GrandioSO Heavy Flow	0.11 ± 0.15 ^A,a,b^	0.10 ± 0.23 ^A,B,a,b^	0.29 ± 0.33 ^A,B,b^	0.34 ± 0.54 ^C,b,c^
Neo-Spectra ST Flow	0.26 ± 0.11 ^A,b^	0.02 ± 0.16 ^B,a,b^	0.15 ± 0.26 ^B,c,b,d^	0.03 ± 0.17 ^B,c,d^
3M Filtek Supreme Flowable	0.24 ± 0.19 ^A,b^	0.00 ± 0.24 ^B,a,b^	0.02 ± 0.12 ^B,a^	0.06 ± 0.09 ^B,c,d^

Abbreviations: SD, standard deviation. Different uppercase superscript letters within the same row indicate statistically significant differences among aging protocols. Different lowercase superscript letters within the same column indicate statistically significant differences among resin composites (*p* < 0.05).

**Table 5:** Mean color change (ΔE*ab) values after immersion in coffee following the aging protocols (mean ± SD).

Resin Composite	Thermocycling	10% Citric Acid	*n*-Heptane	75% Ethanol
G-ænial Universal Injectable	3.16 ± 1.39 ^A,a^	4.69 ± 1.72 ^A,B,a^	5.07 ± 1.93 ^A,B,a^	5.89 ± 1.28 ^B,a,b^
Clearfil Majesty ES Flow	3.18 ± 0.66 ^A,a^	2.55 ± 0.96 ^A,b^	4.61 ± 1.27 ^A,a,c^	7.95 ± 3.24 ^B,b^
Omnichroma Flow	5.46 ± 1.70 ^A,b^	6.11 ± 1.04 ^A,a,c^	7.07 ± 1.15 ^A,b^	7.05 ± 1.13 ^A,a,b^
GrandioSO Heavy Flow	6.94 ± 1.77 ^A,b^	6.85 ± 1.81 ^A,c^	7.32 ± 1.72 ^A,b^	8.41 ± 2.72 ^A,b^
Neo-Spectra ST Flow	4.59 ± 0.96 ^A,b^	6.35 ± 1.60 ^B,a,c^	4.81 ± 1.00 ^B,a,c^	4.66 ± 0.82 ^B,a^
3M Filtek Supreme Flowable	4.29 ± 0.99 ^A,a,b^	4.70 ± 0.83 ^A,a,b^	5.55 ± 1.36 ^A,a,b,c^	4.84 ± 1.64 ^A,a^

Abbreviations: SD, standard deviation. Different uppercase superscript letters within the same row indicate statistically significant differences among aging protocols. Different lowercase superscript letters within the same column indicate statistically significant differences among resin composites (*p* < 0.05).

## Data Availability

The original contributions presented in this study are included in the article. Further inquiries can be directed to the corresponding author.

## Abbreviations

The following abbreviations are used in this manuscript:

ANOVA	Analysis of Variance
CIELAB	Commission Internationale de l’Éclairage L*a*b* Color Space
ΔE*ab	CIELAB Color Difference
ΔRa	Change in Surface Roughness
FSLs	Food-Simulating Liquids
LED	Light-Emitting Diode
Ra	Arithmetic mean Surface Roughness
SD	Standard Deviation

## References

[1] Ferracane J.L. (2011). Resin composite—State of the art. Dent. Mater..

[2] Ilie N., Hickel R. (2011). Resin composite restorative materials. Aust. Dent. J..

[3] Kumari C.M., Bhat K.M., Bansal R., Singh N., Anupama A., Lavanya T. (2019). Evaluation of surface roughness and hardness of newer nanoposterior composite resins after immersion in food-simulating liquids. Contemp. Clin. Dent..

[4] Bollen C.M., Lambrechts P., Quirynen M. (1997). Comparison of surface roughness of oral hard materials to the threshold surface roughness for bacterial plaque retention: A review of the literature. Dent. Mater..

[5] Bagheri R., Burrow M., Tyas M. (2005). Influence of food-simulating solutions and surface finish on susceptibility to staining of aesthetic restorative materials. J. Dent..

[6] Ertas E., Gueler A.U., Yuecel A.C., Koepruelue H., Gueler E. (2006). Color stability of resin composites after immersion in different drinks. Dent. Mater. J..

[7] Ypei Gia N.R., Sampaio C.S., Higashi C., Sakamoto A., Hirata R. (2021). The injectable resin composite restorative technique: A case report. J. Esthet. Restor. Dent..

[8] Sideridou I., Tserki V., Papanastasiou G. (2003). Study of water sorption, solubility and modulus of elasticity of light-cured dimethacrylate-based dental resins. Biomaterials.

[9] Vouvoudi E.C., Sideridou I.D. (2013). Effect of food/oral-simulating liquids on dynamic mechanical thermal properties of dental nanohybrid light-cured resin composites. Dent. Mater..

[10] Kooi T., Tan Q., Yap A., Guo W., Tay K., Soh M. (2012). Effects of food-simulating liquids on surface properties of giomer restoratives. Oper. Dent..

[11] Kakaboura A., Fragouli M., Rahiotis C., Silikas N. (2007). Evaluation of surface characteristics of dental composites using profilometry, scanning electron, atomic force microscopy and gloss-meter. J. Mater. Sci. Mater. Med..

[12] Pala K., Tekce N., Tuncer S., Serim M.E., Demirci M. (2016). Evaluation of the surface hardness, roughness, gloss and color of composites after different finishing/polishing treatments and thermocycling using a multitechnique approach. Dent. Mater. J..

[13] Chowdhury D., Mukherjee S., Maity I., Mazumdar P. (2023). Surface roughness and microhardness evaluation of composite resin restorations subjected to three different polishing systems immediately and after 24 h: An in vitro study. J. Conserv. Dent. Endod..

[14] Sarac D., Sarac Y.S., Kulunk S., Ural C., Kulunk T. (2006). The effect of polishing techniques on the surface roughness and color change of composite resins. J. Prosthet. Dent..

[15] (1997). Geometrical Product Specifications (GPS)—Surface Texture: Profile Method—Terms, Definitions and Surface Texture Parameters.

[16] (1996). Geometrical Product Specifications (GPS)—Surface Texture: Profile Method—Rules and Procedures for the Assessment of Surface Texture.

[17] Wee A.G., Lindsey D.T., Kuo S., Johnston W.M. (2006). Color accuracy of commercial digital cameras for use in dentistry. Dent. Mater..

[18] Guo J., Bing Z., Yang J., Tsoi J.K., Wang Y. (2022). Effect of roughness and acidic medium on wear behavior of dental resin composite. BMC Oral Health.

[19] McKinney J., Wu W. (1985). Chemical softening and wear of dental composites. J. Dent. Res..

[20] Ruivo M.A., Pacheco R.R., Sebold M., Giannini M. (2019). Surface roughness and filler particles characterization of resin-based composites. Microsc. Res. Tech..

[21] Barbieri G.M., Mota E.G., Rodrigues-Junior S.A., Burnett L.H. (2011). Effect of whitening dentifrices on the surface roughness of commercial composites. J. Esthet. Restor. Dent..

[22] Voltarelli F.R., Santos-Daroz C.B.d., Alves M.C., Cavalcanti A.N., Marchi G.M. (2010). Effect of chemical degradation followed by toothbrushing on the surface roughness of restorative composites. J. Appl. Oral Sci..

[23] Yap A., Tan S., Wee S., Lee C., Lim E., Zeng K. (2001). Chemical degradation of composite restoratives. J. Oral Rehabil..

[24] Gale M., Darvell B. (1999). Thermal cycling procedures for laboratory testing of dental restorations. J. Dent..

[25] Ferracane J.L. (2006). Hygroscopic and hydrolytic effects in dental polymer networks. Dent. Mater..

[26] Yilmaz E., Sadeler R. (2016). Effect of thermal cycling and microhardness on roughness of composite restorative materials. J. Restor. Dent..

[27] Checchi V., Generali L., Corciolani L., Breschi L., Mazzitelli C., Maravic T. (2025). Wear and roughness analysis of two highly filled flowable composites. Odontology.

[28] Villalta P., Lu H., Okte Z., Garcia-Godoy F., Powers J.M. (2006). Effects of staining and bleaching on color change of dental composite resins. J. Prosthet. Dent..

[29] Al Kheraif A.A.A., Qasim S.S.B., Ramakrishnaiah R., ur Rehman I. (2013). Effect of different beverages on the color stability and degree of conversion of nano and microhybrid composites. Dent. Mater. J..

[30] Arocha M.A., Mayoral J.R., Lefever D., Mercade M., Basilio J., Roig M. (2013). Color stability of siloranes versus methacrylate-based composites after immersion in staining solutions. Clin. Oral Investig..

[31] Paravina R.D., Ghinea R., Herrera L.J., Bona A.D., Igiel C., Linninger M., Sakai M., Takahashi H., Tashkandi E., Mar Perez M.d. (2015). Color difference thresholds in dentistry. J. Esthet. Restor. Dent..

[32] Patel S.B., Gordan V.V., Barrett A.A., Shen C. (2004). The effect of surface finishing and storage solutions on the color stability of resin-based composites. J. Am. Dent. Assoc..

[33] Guler A.U., Kurt S., Kulunk T. (2005). Effects of various finishing procedures on the staining of provisional restorative materials. J. Prosthet. Dent..

[34] Fonseca A.S.Q., Moreira A.D.L., de Albuquerque P.P.A., de Menezes L.R., Pfeifer C.S., Schneider L.F.J. (2017). Effect of monomer type on the degree of conversion, water sorption and solubility, and color stability of model dental composites. Dent. Mater..

[35] Ardu S., Braut V., Gutemberg D., Krejci I., Dietschi D., Feilzer A.J. (2010). A long-term laboratory test on staining susceptibility of esthetic composite resin materials. Quintessence Int..

[36] Redwan H.S., Hussein M.A., Abdul-Monem M.M. (2025). Effect of Bleaching on Surface Roughness and Color Parameters of Coffee-Stained Nanohybrid Dental Composites with Different Viscosities. Eur. J. Gen. Dent..

[37] Yap A., Tan D., Goh B., Kuah H., Goh M. (2000). Effect of food-simulating liquids on the flexural strength of composite and polyacid-modified composite restoratives. Oper. Dent..

[38] Hahnel S., Henrich A., Bürgers R., Handel G., Rosentritt M. (2010). Investigation of mechanical properties of modern dental composites after artificial aging for one year. Oper. Dent..

[39] Alshali R.Z., Salim N.A., Satterthwaite J.D., Silikas N. (2015). Long-term sorption and solubility of bulk-fill and conventional resin-composites in water and artificial saliva. J. Dent..

[40] Van Noort R., Davis L. (1984). The surface finish of composite resin restorative materials. Br. Dent. J..

[41] Çalışkan M.A., Değirmenci A., Boyacıoğlu H., Turkun S. (2024). Yüksek Dolduruculu Akışkan Kompozit Rezinlerin Çarklı Cila Sistemleri ile Parlatıldıktan Sonraki Yüzey Özellikleri ve Renk Değişimleri. Selcuk Dent. J..

[42] Miletic V. (2018). Dental Composite Materials for Direct Restorations.

[43] Ferracane J.L. (2024). A historical perspective on dental composite restorative materials. J. Funct. Biomater..

[44] Meniawi M., Şirinsükan N., Can E. (2026). Color stability, surface roughness, and surface morphology of universal composites. Odontology.

[45] Freitas F., Pinheiro de Melo T., Delgado A.H., Monteiro P., Rua J., Proença L., Caldeira J., Mano Azul A., Mendes J.J. (2020). Varying the polishing protocol influences the color stability and surface roughness of bulk-fill resin-based composites. J. Funct. Biomater..

